# Supplementary material for: Dynamic transcriptomic profiles of zebrafish gills in response to zinc supplementation
Source: BMC Genomics. 2010 Oct 11;11:553. doi: 10.1186/1471-2164-11-553 (PMC3091702; doi:10.1186/1471-2164-11-553)
Supplement: Additional file 2 — Interactive Direct Interaction Network representing the molecular interactions between zinc, copper, iron, calcium and proteins encoded by transcripts changed by zinc supplementation. Mini web-site containing index.html and hyperlinked pages in subdirectory describing a Direct Interaction Network automatically generated based on curated interactions contained within the proprietary PathwayArchitect database. Ovals represent proteins and the circles symbolize metal ions. Objects are coloured by their abundance in zebrafish at the time-point they were significantly different from the control is a scale from -4 fold (dark green) to +4 fold (dark red). Where significant differences were found at more than one time-point, the colour overlay shows expression at the first instance. Dark blue squares denote 'binding', and light blue squares 'expression'; green squares stand for 'regulation', green diamonds for 'metabolism', and green circles for 'promoter binding'. Arrow heads indicate directionality of the interaction where annotated. All nodes and edges can be further interrogated by selecting the relative area of the image. [file 1471-2164-11-553-S2.zip › PathwayArchitect Zn xs DIN/488359.html]

# REGULATION:

|  |  |
| --- | --- |
| Type | REGULATION |
| Effect | Positive |


---

|  |  |
| --- | --- |
| Score | 0 |


---

|  |  |
| --- | --- |
| Reference Count | 29 |


---

|  |  |
| --- | --- |
| Mechanism | Unknown |


---

|  |  |
| --- | --- |
| Reference:0 || Sentence | "These results suggested that phospholamban regulates the Ca2+ ATPase in dual modes; in short time range, by decreasing the affinity of the Ca2+ ATPase for Ca2+ by phosphorylation of phospholamban with cAMP-dependent protein kinase, and in long time range, by changing the molecular ratio between the two proteins through the regulation of gene expression." |
| PMID | 7815458 |
| Year | 1994 |
| Species | Rat |
| Journal | J Mol Cell Cardiol |
| RefScore | 0 |
| Source | PArchNLP |
  |
|  |  |


---

|  |  |
| --- | --- |
 Reference:1 || Sentence | "Forskolin or PLB-KO increased SR Ca(2+) load, as measured by caffeine-induced Ca(2+) transients." |
| PMID | 11502581 |
| Year | 2001 |
| Species | Mouse |
| Journal | Am J Physiol Cell Physiol |
| RefScore | 1 |
| Source | PArchNLP |
  ||  |  |


---

|  |  |
| --- | --- |
 Reference:2 || Sentence | "Exposure of permeabilized cells to anti-phospholamban antibodies elevated the SR Ca(2+) content and increased the frequency of sparks." |
| PMID | 11463625 |
| Year | 2001 |
| Species | Rat |
| Journal | Biophys J |
| RefScore | 0 |
| Source | PArchNLP |
  ||  |  |


---

|  |  |
| --- | --- |
 Reference:3 || Sentence | "These results indicate that the functional effect of phospholamban phosphorylation is to increase both the Ca2+ sensitivity and the maximum velocity of net Ca2+ uptake into the SR." |
| PMID | 9512486 |
| Year | 1998 |
| Journal | Biochem J |
| RefScore | 1 |
| Source | PArchNLP |
  ||  |  |


---

|  |  |
| --- | --- |
 Reference:4 || Sentence | "Studies in animal models have suggested that alterations affecting phospholamban-mediated stimulation of Ca2+ uptake by sarcoplasmic reticulum are involved in the pathophysiology of heart disease." |
| PMID | 2139670 |
| Year | 1990 |
| Species | Human |
| Journal | J Clin Invest |
| RefScore | 0 |
| Source | PArchNLP |
  ||  |  |


---

|  |  |
| --- | --- |
 Reference:5 || Sentence | "Stimulation of Ca2+ uptake by anti-phospholamban monoclonal antibody simulated the effect of phosphorylation of phospholamban by cAMP-dependent protein kinase." |
| PMID | 2139670 |
| Year | 1990 |
| Species | Human |
| Journal | J Clin Invest |
| RefScore | 0 |
| Source | PArchNLP |
  ||  |  |


---

|  |  |
| --- | --- |
 Reference:6 || Sentence | "Phospholamban-mediated stimulation of Ca2+ uptake in human cardiac sarcoplasmic reticulum is thus comparable in magnitude to that observed in other species and results from an increase in the apparent affinity of the Ca2(+)-transporting ATPase for Ca2+." |
| PMID | 2139670 |
| Year | 1990 |
| Species | Human |
| Journal | J Clin Invest |
| RefScore | 1 |
| Source | PArchNLP |
  ||  |  |


---

|  |  |
| --- | --- |
 Reference:7 || Sentence | "When five of these monoclonal antibodies were assessed for their ability to affect SR Ca2+ uptake three of these antibodies had no effect on SR Ca2+ uptake, whereas the other two monoclonals were able to stimulate SR Ca2+ uptake to levels similar to those caused by phosphorylation of phospholamban at different calcium concentrations." |
| PMID | 1710223 |
| Year | 1991 |
| Journal | J Biol Chem |
| RefScore | 1 |
| Source | PArchNLP |
  ||  |  |


---

|  |  |
| --- | --- |
 Reference:8 || Sentence | "Therefore, in human myocardium, the Ca(2+)-sensitivity but not the Vmax of SERCA 2a is regulated by cAMP-dependent phosphorylation of phospholamban at position serine-16." |
| PMID | 10198180 |
| Year | 1999 |
| Species | Human |
| Journal | J Mol Cell Cardiol |
| RefScore | 1 |
| Source | PArchNLP |
  ||  |  |


---

|  |  |
| --- | --- |
 Reference:9 || Sentence | "These findings suggest that although decreased SERCA protein and phospholamban phosphorylation levels contribute to depressed SR Ca2+-uptake and left ventricular function in heart failure, the specific subcellular alterations which underlie these effects may not be uniform with respect to gender." |
| PMID | 11437540 |
| Year | 2001 |
| Species | Human |
| Journal | J Mol Cell Cardiol |
| RefScore | 1 |
| Source | PArchNLP |
  ||  |  |


---

|  |  |
| --- | --- |
 Reference:10 || Sentence | "Isoproterenol perfusion of fetal hearts had no significant effect on their relaxation parameters or on the EC50 of the Ca(2+)-pump for Ca2+, consistent with the low relative levels of phospholamban expressed in these hearts." |
| PMID | 8523443 |
| Year | 1995 |
| Journal | J Mol Cell Cardiol |
| RefScore | 1 |
| Source | PArchNLP |
  ||  |  |


---

|  |  |
| --- | --- |
 Reference:11 || Sentence | "We found no direct effect of the Pl Ab on Ca2+ binding by the ATPase in the absence of ATP." |
| PMID | 8349590 |
| Year | 1993 |
| Journal | J Biol Chem |
| RefScore | 1 |
| Source | PArchNLP |
  ||  |  |


---

|  |  |
| --- | --- |
 Reference:12 || Sentence | "Therefore, Ab binding to Pl reduces specifically the activation energy for a slow transition triggered by Ca2+ binding, with consequent enhancement of overall kinetics under conditions enhancing the rate-limiting contribution of this transition." |
| PMID | 8349590 |
| Year | 1993 |
| Journal | J Biol Chem |
| RefScore | 0 |
| Source | PArchNLP |
  ||  |  |


---

|  |  |
| --- | --- |
 Reference:13 || Sentence | "The reduced phosphorylation state of PLB may lead to decreased Ca2+ sensitivity of SERCA2 in failing human myocardium." |
| PMID | 10603952 |
| Year | 1998 |
| Species | Human |
| Journal | Ann N Y Acad Sci |
| RefScore | 0 |
| Source | PArchNLP |
  ||  |  |


---

|  |  |
| --- | --- |
 Reference:14 || Sentence | "Phospholamban phosphorylation, in pmoles/mg of SR protein, increased from control values, 25 +/- 6 to 110 +/- 11 (ISO-NI-FE), 117 +/- 17 (ISO-Ca+2) and 197 +/- 21 (ISO) and did not change with high calcium." |
| PMID | 3188961 |
| Year | 1988 |
| Species | Rat |
| Journal | Acta Physiol Pharmacol Latinoam |
| RefScore | 1 |
| Source | PArchNLP |
  ||  |  |


---

|  |  |
| --- | --- |
 Reference:15 || Sentence | "The expression of sarcoplasmic reticulum (SR)-Ca(2+)-ATPase and phospholamban, proteins involved in the Ca(2+) uptake of the SR, were only increased in CSQ, indicating a possible influence of NCX in the regulation of SR-Ca(2+) uptake proteins." |
| PMID | 10900244 |
| Year | 2000 |
| Species | Mouse |
| Journal | J Pharmacol Exp Ther |
| RefScore | 1 |
| Source | PArchNLP |
  ||  |  |


---

|  |  |
| --- | --- |
 Reference:16 || Sentence | "These findings indicate that adenovirus-mediated gene transfer of phospholamban modifies intracellular Ca2+ handling and the frequency response in cardiomyocytes." |
| PMID | 9242175 |
| Year | 1997 |
| Species | Rat |
| Journal | Circ Res |
| RefScore | 0 |
| Source | PArchNLP |
  ||  |  |


---

|  |  |
| --- | --- |
 Reference:17 || Sentence | "Stimulation of Ca(2+) uptake by PKA-catalyzed PLB phosphorylation was weaker in diabetic than in control SR vesicles." |
| PMID | 14691046 |
| Year | 2003 |
| Species | Rat |
| Journal | Br J Pharmacol |
| RefScore | 1 |
| Source | PArchNLP |
  ||  |  |


---

|  |  |
| --- | --- |
 Reference:18 || Sentence | "The increase in the apparent affinity of the Ca pump for Ca2+ induced by PLB phosphorylation was clearly reduced at high ionic strength, i.e. under conditions known to reduce the surface membrane potential and all processes dependent on it." |
| PMID | 2538350 |
| Year | 1989 |
| Journal | FEBS Lett |
| RefScore | 1 |
| Source | PArchNLP |
  ||  |  |


---

|  |  |
| --- | --- |
 Reference:19 || Sentence | "These findings suggest that phospholamban is a major regulator of the affinity of Ca(2+)-ATPase for Ca2+, and coordinate regulation of the expression levels of these two SR proteins may be necessary for maintaining Ca2+ homeostasis in the developing mammalian heart." |
| PMID | 9038922 |
| Year | 1997 |
| Species | Mouse |
| Journal | Am J Physiol |
| RefScore | 1 |
| Source | PArchNLP |
  ||  |  |


---

|  |  |
| --- | --- |
 Reference:20 || Sentence | "This review summarizes findings in murine genetic models with alterations in the expression levels of the sarcolemmal Ca(2+)-ATPase and Na+/Ca2+ exchanger, which move Ca2+ across the cell membrane, and the sarcoplasmic reticulum proteins, which are involved in Ca2+ sequestration (Ca(2+)-ATPase and its regulator, phospholamban), Ca2+ storage (calsequestrin), and Ca2+ release (ryanodine receptor, FK506-binding protein and junctin) during excitation-contraction coupling." |
| PMID | 10845094 |
| Year | 2000 |
| Species | Human |
| Journal | Annu Rev Physiol |
| RefScore | 1 |
| Source | PArchNLP |
  ||  |  |


---

|  |  |
| --- | --- |
 Reference:21 || Sentence | "Relative efficiencies of antibodies in Ca2+ pump stimulation and on phospholamban phosphorylation were not correlated." |
| PMID | 2955797 |
| Year | 1987 |
| Species | Mouse |
| Journal | Biochem Cell Biol |
| RefScore | 0 |
| Source | PArchNLP |
  ||  |  |


---

|  |  |
| --- | --- |
 Reference:22 || Sentence | "Isoproterenol stimulation was associated with increases in the affinity of the sarcoplasmic reticulum Ca2+ pump for Ca2+ that were due to phospholamban phosphorylation." |
| PMID | 1394867 |
| Year | 1992 |
| Species | Mouse |
| Journal | Circ Res |
| RefScore | 1 |
| Source | PArchNLP |
  ||  |  |


---

|  |  |
| --- | --- |
 Reference:23 || Sentence | "Phospholamban (PLB) ablation is associated with enhanced sarcoplasmic reticulum (SR) Ca2+ uptake and attenuation of the cardiac contractile responses to beta-adrenergic agonists." |
| PMID | 9374653 |
| Year | 1997 |
| Species | Mouse |
| Journal | Am J Physiol |
| RefScore | 2 |
| Source | PArchNLP |
  ||  |  |


---

|  |  |
| --- | --- |
 Reference:24 || Sentence | "The present study investigated the ontogenetic expression of SR-associated CaM kinase in the rabbit myocardium as well as development-related changes in CaM kinase-mediated phosphorylation of the SR proteins (Ca2+ pump, Ca2+ release channel and phospholamban) involved in transmembrane Ca2+ cycling." |
| PMID | 9040054 |
| Year | 1997 |
| Journal | J Mol Cell Cardiol |
| RefScore | 1 |
| Source | PArchNLP |
  ||  |  |


---

|  |  |
| --- | --- |
 Reference:25 || Sentence | "The Ca(2+)-dependence of the ATPase activity of this reconstituted preparation is virtually identical with that observed using native cardiac SR before and after PLB phosphorylation, indicating that co-reconstituted sarcoplasmic/endoplasmic-reticulum Ca(2+)-ATPase 1 (SERCA1) and PLB provide an equivalent experimental model for SERCA2a-PLB interactions." |
| PMID | 10998362 |
| Year | 2000 |
| Journal | Biochem J |
| RefScore | 1 |
| Source | PArchNLP |
  ||  |  |


---

|  |  |
| --- | --- |
 Reference:26 || Sentence | "Phosphorylation of phospholamban (PLB) at Ser16 (protein kinase A site) and at Thr17 [Ca2+/calmodulin kinase II (CaMKII) site] increases sarcoplasmic reticulum Ca2+ uptake and myocardial contractility and relaxation." |
| PMID | 12763747 |
| Year | 2003 |
| Species | Rat |
|  | Mouse |
| Journal | Am J Physiol Heart Circ Physiol |
| RefScore | 0 |
| Source | PArchNLP |
  ||  |  |


---

|  |  |
| --- | --- |
 Reference:27 || Sentence | "Phospholamban, an endogenous inhibitor of sarcoplasmic reticulum Ca2+ ATPase in the sarcoplasmic reticulum, plays an important role in Ca2+ cycling in heart." |
| PMID | 12645548 |
| Year | 2003 |
| Species | Mouse |
|  | Human |
| Journal | J Biol Chem |
| RefScore | 0 |
| Source | PArchNLP |
  ||  |  |


---

|  |  |
| --- | --- |
 Reference:28 || Sentence | Stimulation of Ca(2+) uptake by PKA-catalyzed PLB phosphorylation was weaker in diabetic than in control SR vesicles. |
| Year | 2004 |
| PMID | 14691046 |
| Species | Rat |
| Journal | Br J Pharmacol |
| RefScore | 1 |
| Source | PArchNLP |
  |


---

|  |  |
| --- | --- |
